# Efferocytosis is restricted by axon guidance molecule EphA4 via ERK/Stat6/Mertk signaling following brain injury

**DOI:** 10.21203/rs.3.rs-3079466/v1

**Published:** 2023-06-27

**Authors:** Eman Soliman, John Leonard, Erwin Kristobal Basso, Ilana Gershenson, Jing Ju, Jatia Mills, Caroline Jager, Alexandra M. Kaloss, Mohamed Elhassanny, Daniela Pereira, Michael Chen, Xia Wang, Michelle H. Theus

**Affiliations:** Virginia Tech; Virginia Tech; Virginia Tech; Virginia Tech; Virginia Tech; Virginia Tech; Virginia Tech; Virginia Tech; Virginia Tech; Virginia Tech; Virginia Tech; Virginia Tech; Virginia Tech

**Keywords:** Efferocytosis, Neuroinflammation, Traumatic brain injury, Apoptosis, Peripheral-derived macrophages, Microglia, Eph, Ephrin, Mertk

## Abstract

**Background:**

Efferocytosis is a process that removes apoptotic cells and cellular debris. Clearance of these cells alleviates neuroinflammation and prevents the release of inflammatory molecules and promotes the production of anti-inflammatory cytokines to help maintain tissue homeostasis. The underlying mechanisms by which this occurs in the brain after injury remains ill-defined.

**Methods:**

We demonstrate using GFP bone marrow chimeric knockout (KO) mice, that the axon guidance molecule EphA4 receptor tyrosine kinase is involved in suppressing Mertk signaling in the brain to restrict the function of efferocytosis on resident microglia and peripheral-derived monocyte/macrophages.

**Results:**

Single-cell RNAseq identified Mertk expression, the primary receptor involved in efferocytosis, on monocytes, microglia, and a subset of astrocytes in the damaged cortex following brain injury. Loss of EphA4 on infiltrating GFP-expressing immune cells improved functional outcome concomitant with enhanced efferocytosis, and overall protein expression of p-Mertk, p-ERK, and p-Stat6. The percentage of GFP^+^ monocyte/macrophages and resident microglia engulfing NeuN^+^ or TUNEL^+^ cells was significantly higher in KO chimeric mice. Importantly, mRNA expression of *Mertk* and its cognate ligand *Gas6* was significantly elevated in these mice compared to wild-type. Analysis of cell-specific expression showed that p-ERK and p-Stat6 co-localized with Mertk-expressing GFP + cells in the peri-lesional area of the cortex following brain injury. Using an *in vitro* efferocytosis assay, co-culturing pHrodo-labeled apoptotic Jurkat cells and bone marrow (BM)-derived macrophages, we demonstrate that efferocytosis efficiency and mRNA expression of *Mertk* and *Gas6* was enhanced in the absence of EphA4. Select inhibitors of ERK and Stat6 attenuated this effect confirming that EphA4 suppresses monocyte/macrophage efferocytosis via inhibition of the ERK/Stat6 pathway.

**Conclusions:**

Our findings implicate the Mertk/ERK/Stat6 axis as a novel regulator of apoptotic debris clearance in brain injury that is restricted by peripheral myeloid-derived EphA4 to prevent the resolution of inflammation.

## Background

Traumatic brain injury (TBI) is a major cause of morbidity and disability worldwide [1]. The secondary injury cascade that follows a traumatic event is mainly triggered by neuroinflammation. Neuroinflammation is the primary driver of secondary injury, with microglia and peripheral-derived macrophages (PDMs) playing critical roles in regulating the production of pro-inflammatory mediators [2]. These two ontogenically distinct phagocytes mediate neuronal dysfunction through the production of inflammatory and cytotoxic molecules, while also promoting neuroprotection through efferocytosis, the process of clearing apoptotic cell debris formed after tissue damage. [3, 4] [5].

Efferocytosis is a complex, well-orchestrated process to remove apoptotic cell debris by phagocytes to enable the resolution of inflammation and tissue repair. However, a prolonged or excessive inflammatory response reduces efferocytosis efficiency and delays tissue recovery [5]. Recognition and engulfment of apoptotic cells are mediated by a network of interactions, including “find-me”, “eat-me”, and engulfment signals. During inflammation, apoptotic cells secrete molecules serving as “find-me” signals that attract phagocytes to eliminate the corpses. The cell surface expression of “eat-me” signals ensures the recognition of apoptotic cells by phagocytes. Phosphatidyl serine (PtdSer) is the most recognized “eat-me” signal expressed on the surface of apoptotic cells, which binds directly or indirectly to phagocytic receptors to initiate the engulfment. The indirect binding of PtdSer to the phagocytic receptors is mediated by engulfment signals secreted by phagocytes. PtdSer/(Gas6 or Pros1)/Mertk are well-characterized binding complexes for phagocyte efferocytosis, including macrophages and microglia. Although apoptosis is a hallmark of brain trauma and a determinant of the severity of TBI outcomes [6–8], the mechanisms regulating efferocytosis following injury remains ill-defined

The erythropoietin-producing hepatocellular carcinoma receptors type A (EphA) are tyrosine kinase receptors which, upon activation by cell surface-bound ephrin ligands, initiate bidirectional signaling implicated in neurodevelopment [9]. Following brain trauma, EphA4 is overexpressed in the damaged cortical tissues, and global deficiency of this receptor demonstrates neuroprotection [10]. In prior work, we find EphA4 is upregulated on both microglia and PDMs. Interestingly, conditional deletion of EphA4 in resident microglia failed to improve TBI outcomes [11]; however, peripheral EphA4 deficiency reduced neuroinflammation and showed neuroprotection [12]. The current study reveals a key mechanism by which peripheral-derived EphA4 limits the innate immune response to resolving tissue inflammation in the brain. This is the first evidence that the process of efferocytosis occurs in a limited fashion in the brain following a traumatic insult and that enhancing this process via targeting Mertk/EphA4 may be a novel strategy to improve outcomes.

## Materials and methods

### Animals.

Male *Cx3cr1*^*CreER /+*^, *EphA4*^f/f^, *Epha4*^+/+^/*ROSA*^*mTmG*^*/Tie2-Cre*, and *Epha4*^f/f^/ ROSA^mTmG^/Tie2-Cre mice were housed in an AAALAC-accredited animal facility with a 12-h light-dark cycle and food and water provided ad libitum. Animal experiments were performed following the NIH Guide for the Care and Use of Laboratory Animals and approved by the Virginia Tech Institutional Animal Care and Use Committee (IACUC; #21–044). *Epha4*^f/f^ recipient mice and *Epha4*^*+/+*^*/ROSA*^*mTmG*^*/Tie2-Cre* and *Epha4*^f/f^/*ROSA*^*mTmG*^*/Tie2-Cre* donor mice used for the chimeric generation were bred and genotyped as previously described [13–15].

### Generation of bone marrow chimeric mice.

Bone marrow ablation was induced by exposing *EphA4*^*f/f*^ recipient mice (6–8 weeks) to X-ray irradiation (two doses of 550 rad at six h apart). Within 24 h of irradiation, recipient mice were intravenously injected with 3–4 million bone marrow cells (BMCs) isolated from femur and tibia of donor *Rosa26*^*mtmg*^ */Tie2-Cre/EphA4*^*+/+*^*or Rosa26*^*mtmg*^*/Tie2-Cre/EphA4*^*f/f*^ mice as previously described [10, 12]. Recipient mice were placed on gentamycin sulfate water (1 mg/ml) for three days before irradiation and two weeks after adoptive transfer. Controlled cortical impact (CCI) injury for adoptive transfer WT (WT^+ WTBMCs^) and KO (WT^+ KOBMCs^) was performed at four weeks post-chimera generation.

### Controlled cortical impact injury.

CCI injury was induced as previously described [10, 11]. Briefly, mice were anesthetized with subcutaneous ketamine (100 mg/kg) and xylazine (10 mg/kg) and securely positioned in a stereotaxic frame, maintaining a body temperature of 37°C. A midline incision was made on the sanitized and shaved scalp to expose the skull. Then, a craniectomy (4 mm diameter) was performed over the right parietal-temporal cortex (− 2.5 mm A/P and 2.0 mm lateral from the bregma) using a portable drill. The injury was induced with a velocity of 5 m/s and a depth of 2 mm. The incision was sutured, and animals were monitored until recovery.

### Perfusion fixation and brain serial sectioning.

At 1- or 3-days post-CCI injury (dpi), mice were euthanized by subcutaneously (s.c.) injecting a combination of 150 mg/kg ketamine and 20 mg/kg xylazine and perfusion fixation was performed as previously described [11]. Five minutes before perfusion, heparin (2000 units/kg, s.c.) and sodium nitroprusside (0.75 mg/kg, s.c.) were injected. After confirming the loss of pedal reflex, cardiac perfusion of heparin (20 units/ml) in phosphate-buffered saline (PBS) was performed to clear the circulation of blood, followed by perfusion with ice-cold 4% paraformaldehyde (PFA) in PBS. Fixed brains were cryoprotected with gradient sucrose solutions, snap-frozen, embedded in OCT compound with 30% sucrose, and stored at −80°C until sectioning. Serial coronal sections (30 μm) were obtained using a cryostat (− 1.1 to − 2.6 mm posterior from bregma), mounted on positively charged slides (with five sections spaced 450 μm apart), and stored at −80°C for further analysis.

### Single-cell sequencing, library generation, Gene Ontology enrichment, and computational analyses.

At 1-day post-CCI injury, CD1 mice were euthanized using isoflurane and 4 mm x 4mm ipsilateral (injured) cortical tissues were harvested and dissociated using papain digest neural dissociation kit (Miltenyi Biotech). Cells were then cryopreserved in 1mL of CryoStore^®^ CS10 media (Stem cell technologies, Seattle, WA, USA) and sent to Medgenome for scRNAseq (Foster City, California, USA). ScRNA-seq libraries were generated using the Chromium Next GEM Single Cell 5’ v2 chemistry (10x Genomics) and sequenced on a NovaSeq 6000 (Illumina). An alignment of the libraries and read counts were performed using mouse RNA-Seq Database consisting of expression values of 358 bulk Mouse RNA-seq samples of sorted cell populations (Cell Ranger V7.0, 10X Genomics). Quality control was performed excluding the genes that are not expressed in at least 3 cells and the cells that do not express > 200 genes (Seurat, V 4.1.0 Read10X function). Doublet Finder package, v2.0.3 was used to filter for doublets, LogNormalize was used for global-scaling normalization, and Seurat was used for clustering. Unbiased cell type recognition from scRNAseq data was performed using Cellenics. The following markers were used for the clustering of endothelial cells (Cd31, Tie2, Cdh5, Glut1), microglia (Tmem119, Ccr2-, cx3cr1), astrocytes (GFAP, Aldhl1, sox9), and monocytes/macrophages (Ccr2, Mmp8) as well as program automated clusters by brain tissue type. We applied modularity optimization by Louvain algorithm to iteratively group cells together and visualize the data using UMAP.

### Quantitative real-time PCR.

Total RNA was freshly isolated from cultured bone marrow-derived macrophages, ipsilateral (4×4 mm), or contralateral (4×4 mm) cortical tissue using TRIzol^®^ reagent (Ambion) according to manufacturer’s instructions. RNA concentration was measured using a spectrophotometer ND-1000 (NanoDrop). DNAse I treatment (for a 1000 ng RNA) was performed to degrade the genomic DNA using an amplification grade Kit (Sigma Aldrich, St. Lois, MO). To synthesize cDNA, reverse transcription was performed using iScript^™^ cDNA synthesis kit (Biorad, Hercules, CA). For qRT-PCR, iTaq^™^ Universal SYBR^®^ Green Supermix (Biorad, Hercules, CA) and specific primers (Table 1) were used to amplify cDNA (50 ng) following the manufacturer’s instructions. mRNA expression (fold change) was calculated relative to GAPDH as an internal control using 2^−ΔΔCT^ method.

### Immunohistochemistry and confocal image analysis.

Serial coronal sections were washed in PBS, subsequently blocked 2% cold water fish gelatin/0.2% Triton X-100 (Sigma, Inc.) for 2 hours, and then incubated overnight with the following primary antibodies diluted in blocking solutions: Rb anti-IBA1 (Wako) antibody (1:250), Rt anti-IBA1 (Abcam) antibody (1:250), Rt anti-Mertk (ThermoFisher) antibody (1:200), Rb anti-Mertk (Abcam) antibody (1:200), Rb anti-NeuN (cell signaling) antibody (1:200), Rb anti-P-ERK (cell signaling) antibody (1:200), or Rb anti-P-Stat6 (cell signaling) antibody (1:150). Afterward, the sections were washed with 1× PBS and incubated with the appropriate secondary antibodies (Invitrogen) diluted (1:250) in the blocking solution for 1 hour: AlexFluor donkey anti-rabbit-594, AlexFluor donkey anti-rat-647, AlexFluor donkey anti-rat-594, or AlexFluor donkey anti-rabbit-647. Following another round of washing with 1× PBS, the sections were mounted using a media containing DAPI counterstain (SouthernBiotech). Confocal images were acquired using a Zeiss 880 confocal microscope (Carl Zeiss, Oberkochen, Germany).

### Stereological cell counts.

Cell quantification was assessed by a blinded investigator using the optical fractionator probe function of Stereoinvestigator (MicroBrightField, Williston, VT, USA) on an upright Olympus BX51TRF motorized microscope (Olympus America, Center Valley, PA, USA) with a grid size set at 450 × 450 mm and 150 × 150 mm counting frame for cortex as previously described [16, 17].

### TUNEL staining/imaging and counting.

TUNEL staining of serial coronal sections of perfused fixed brains was performed using the Click-iT Plus TUNEL Assay; 647 (Thermo Fisher Scientific) according to the manufacturer’s instructions. Serial coronal sections (300 μm) were permeabilized with 0.2% Triton X-100 in phosphate-buffered saline (PBS) and incubated with the TUNEL reaction mixture containing terminal deoxynucleotidyl transferase enzyme followed by Alexa Fluor 647-conjugated nucleotides. Following the TUNEL reaction, the sections were blocked in 2% cold water fish gelatin (Sigma, Inc.) in 0.2% triton for 1 h, then incubated with Rb anti-IBA1 (Wako) or Rb anti-NeuN (cell signaling) antibody (1:200 in blocking solution) overnight. Slides were then washed with 1×PBS, treated with AlexFluor donkey anti-rabbit-555 (1:250 in block) for 1 hour, further washed in 1×PBS, and then mounted in media with DAPI counterstain (SouthernBiotech). Z-stack images were acquired using a Zeiss 880 confocal microscope (Carl-Zeiss, Oberkochen, Germany).

### Western blot analysis-

At three days post-CCI injury, mice were euthanized using isoflurane and 4 mm x 4mm ipsilateral (injured) or contralateral cortical tissues were harvested and freshly homogenized in RIPA lysis buffer (Pierce, ThermoFisher) containing protease/phosphatase inhibitor cocktail mix (Halt, ThermoFisher). Tissue lysate was centrifuged at 12,000 rpm for 20 min and protein concentration was determined using BCA reagents (Thermo Fisher Scientific, Inc., Rockford, IL). Equal protein concentrations of each sample were loaded onto SDS–PAGE gels and transferred to PVDF (BioRad, Hercules, CA) using transfer buffer (containing 190 mM glycine, 25 mM Tris, 0.5% SDS, and 20% methanol). Membranes were blocked at room temperature using Intercept^®^ (TBS) blocking buffer (LI-COR Biosciences) for two hours and then incubated overnight with the following primary antibodies (1:1000 in blocking solution): Rb anti-Phosphorylated Mertk (PhosphSolutions, Aurora, CO), Rb anti-Mertk (Abcam), Rb anti-Phosphorylated ERK 1/2 (Cell signaling), Ms anti-ERK 1/2 (Cell signaling), Rb anti-Phosphorylated Stat6 (Cell signaling), Rb anti-Stat6 (Cell signaling), and Ms anti-actin (Cell signaling). Membranes were washed with TBST and then incubated with LICOR IRDye anti-rabbit and/or anti-mouse IgG (LI-COR Biosciences) for one hour. Protein bands were imaged with LI-COR Odyssey system and quantified using Fiji Image-J software.

### In vitro efferocytosis assay.

**Bone marrow-derived macrophages (BMDMS) culturing and treatment-** GFP + Bone marrow-derived cells (BMCs) were isolated from the femurs of EphA4-WT (Rosa26mtmg /Tie2-Cre/EphA4+/+) or EphA4-KO (Rosa26 mtmg/Tie2-Cre/EphA4f/f) mice and cultured (1×10^6^ cells/ml) in complete RPMI medium with fetal bovine serum (10%), L-glutamine (2 mM), penicillin/streptomycin (1%), and M-CSF (10 ng/ml) as previously described [10]. On day 5, bone marrow-derived macrophages (BMDMS) were supplemented with fresh RPMI media or pretreated with 1 μg/ml of Escherichia coli O111:B4 LPS (Sigma Aldrich, St. Louis, MO) for 4 hours, 0.5 μg/ml of mouse recombinant HMGB1 (ThermoFisher Scientific) for 4 hours, 5 μg/ml of EphA4-Fc or Fc-control clustered using 1.7 μg/ml α-Fc (Sino Biological, Wayne, PA) for 1 hour, 25 μM of ERK inhibitor (FR18020R, Cayman chemicals) for 4 hours, 5 μM of Mertk inhibitor (UNC2025, Cayman Chemicals) for 4 hours, or 250 nM of Stat6 inhibitor (AS1517499, Cayman Chemicals) for 4 hours. **Jurkat cells culturing and treatment-** Jurkat cells were cultured in complete RPMI medium, counted daily, and cell density was maintained between 2×10^5^ and 1 × 10^6^ cells/ml until a sufficient number of cells was obtained as required for experiments. On the day of the efferocytosis experiment, Jurkat cells 1 × 10^6^ cells/ml were treated with 1 μM of Staurosporine (STS, Cayman chemical) for 3 hours. Apoptosis was confirmed using Alexafluor 488 Annexin/dead cells apoptosis kit (Invitrogen) containing Annexin V (AnnV) and Propidium Iodide (PI) per manufacturer’s instructions. ImageStream Flow cytometry was used to measure early apoptotic (AnnV+/PI−) and late apoptotic/necrotic (AnnV+/PI+) cells. More than 90% of apoptotic (both early and late) cells were required for the engulfment assay. After STS treatment, apoptotic Jurkat cells were collected, washed with HBSS, then stained with 0.5uM pHrodo^™^ Red succinimidyl (NHS) ester (ThermoFisher). Stained live and apoptotic Jurkat cells were washed, resuspended in complete RPMI media, and added to the previously cultured BMDMS in a 10:1 ratio. Cells were incubated for 1 hour then the non-engulfed Jurkat cells were washed off with ice-cold PBS. Adherent GFP + BMDMS engulfing pHrodo + apoptotic Jurkat cells were fixed with ice-cold 4% PFA and counterstained with DAPI. The total number of BMDMS and BMDMS engulfing apoptotic Jurkats were counted, and the efferocytosis efficiency (BMDMS engulfing apoptotic Jurkat cells/total number of BMDMS x 100) was calculated as previously described [18].

### Cerebral blood flow analysis.

A laser speckle contrast imaging system (RFLSI III Laser Speckle Imaging System, RWD Life Science, Dover, DE, USA) was used to scan cerebral blood flow over the right parietal cortex after performing a craniectomy (4 mm diameter). Cerebral blood flow was recorded in the region of interest (ROI, 2.5 mm diameter) for 30 seconds and 4 readings for each recording were taken before injury (baseline), at 10 min, and 3 dpi. The average of the 4 readings and the percentage from the baseline were calculated for each mouse. Laser speckle contrast (LSC) and bright field images were captured at every recording.

### Blood-brain barrier permeability (IgG deposition).

Serial coronal sections of perfused fixed brains were blocked in 2% cold water fish gelatin (Sigma, Inc.) in 0.2% triton for 1 hour, incubated with AlexFluor donkey anti-mouse-594 (1:250 in block) for 1 hour, washed in 1×PBS, and then mounted in media with DAPI counterstain (SouthernBiotech). The volume of IgG deposition (mm^3^) was measured using the Cavalieri Estimator from StereoInvestigator software.

### Statistical Analysis.

Data are presented as mean ± standard error of the mean (SEM), graphed, and analyzed using the GraphPad Prism program, version 9 (GraphPad Software, Inc., San Diego, CA). The intergroup variations were analyzed using Student’s unpaired two-tailed t-test (to compare between two experimental groups), one-way analysis of variance (ANOVA) followed by Bonferroni’s multiple comparisons test (to compare multiple groups with one independent variable), or two-way ANOVA followed by Šídák’s multiple comparisons test (to compare multiple groups with two independent variables). The variations were considered significant at P < 0.05.

## Results

### Efferocytosis-related gene expression across single cells in the damaged cortex.

**Figure 1:**
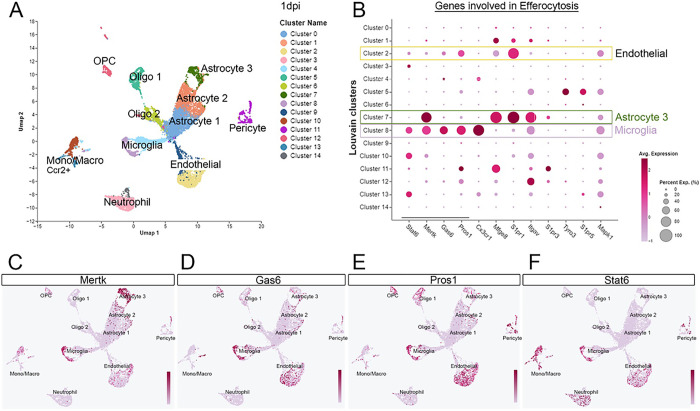
ScRNAseq analysis displays a differential expression of efferocytosis signals in the brain following CCI injury. (A) Uniform Manifold Approximation and Projection (UMAP) plot showing different cell clusters and their cell-specific annotation in the injured cortex at 1dpi (B) Dot plot of efferocytosis genes expressed by each cluster type. (C-F) Feature maps highlighting the expression across clusters of Mertk (C), Gas 6 (D), Pros1 (E), and Stat6 (F).

Efferocytosis has not been fully characterized in brain injury as a result of trauma. To identify specific cell types across the damaged cortex that expression efferocytosis-related genes, we conducted scRNA-seq expression analysis on cells dissociated from the ipsilateral cortex at 1-day post-CCI injury. A total of 5000 cells were included in our analysis. Uniform Manifold Approximation and Projection (UMAP) plot shows different clusters of microglia, astrocytes, endothelial cells, pericytes, neutrophils, and monocytes/macrophages isolated from the injured cortex based on RNA gene expression (Fig. 1A). The analysis of differential expression of efferocytosis signals in different cell clusters revealed the expression of efferocytic receptor, *Mertk*, is present predominately in microglia and astrocytes, as well as monocyte/macrophages and endothelial cells. Its cognate ligands, *Gas6* and *Pros1*, predominate in microglia and endothelial cells (Fig. 1B). Feature UMAPs highlight the spatial location of these genes in the different clusters confirming their enriched expression (Fig. 1C–1F).

### Upregulation of Mertk in the ipsilateral cortex following CCI injury.

**Figure 2:**
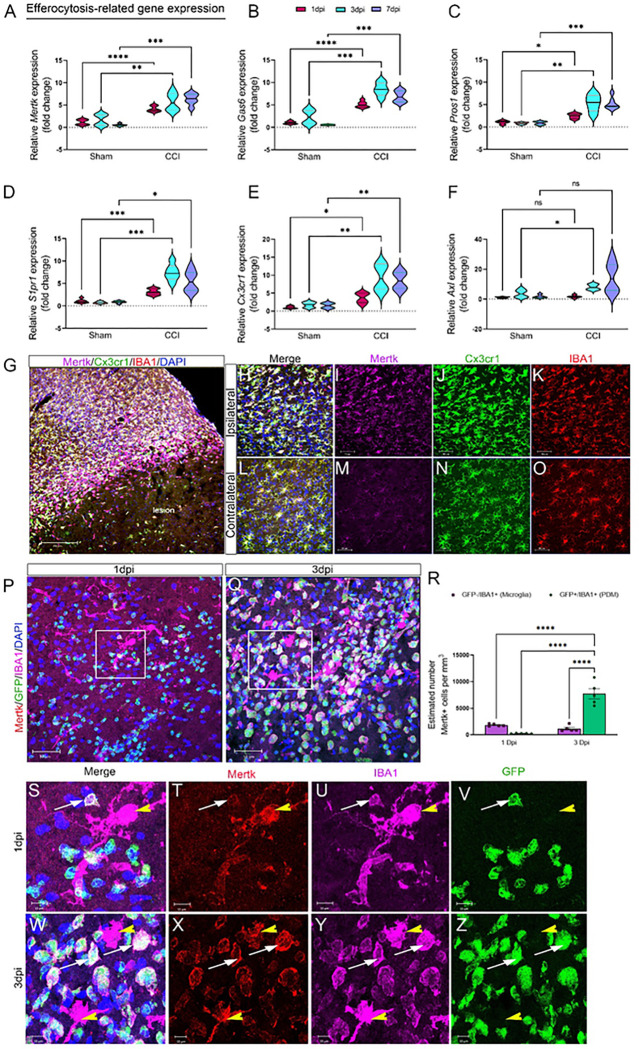
Efferocytosis-related gene changes and Mertk expression in the damaged cortex following CCI injury. A-F) Relative mRNA expression of Mertk (A), Gas6 (B), Pros1 (C), S1pr1 (D), Cx3cr1 (E), and Axl (F) in the ipsilateral cortex of CCI-injured wild type mice at 1, 3, and 7 dpi. G-O) Representative confocal images for Mertk expression in the wild-type *Cx3cr1* ^*EYFP-CreER /+*^ mice at 3 dpi. G) 2×2 tile confocal images for the ipsilateral cortex showing Mertk (purple), Cx3cr1 (green), and IBA1 (red) expression. Mertk is upregulated and colocalized with Cx3cr1 and IBA1 in the ipsilateral cortex (H-K). Mertk expression is low in Cx3cr1+IBA1+ cells in the contralateral cortex (L-O). P-Z) Mertk expression in microglia and PDM in the ipsilateral cortex of CCI-injured GFP^+^ bone marrow chimeric wild type (WT^+WTBMCs^) mice. Mertk (red) is upregulated in GFP^−^ IBA1^+^ microglia at 1dpi (P& S-V) and 3dpi (O &W-Z) and in GFP^+^IBA1^+^ PDM at 3 dpi (O& W-Z). The total number of Mertk^+^GFP^−^IBA1^+^ microglia and Mertk^+^GFP^+^IBA1^+^ PDM was counted in the ipsilateral cortex at 1- and 3- dpi using the optical fractionator probe function of Stereoinvestigator. N=5–6 mice/group. *P<0.05; **P<0.01; ***P<0.001, ****P<0.0001. Two-way ANOVA followed by Šídák’s multiple comparisons test.

To investigate the effect of CCI injury on efferocytosis-related genes, we measured temporal mRNA expression of receptors involved in apoptotic cell recognition and engulfment in the ipsilateral cortex at 1, 3, and 7dpi. We observed a significant increase in recognition receptors (*S1pr1* and *Cx3cr1*), engulfment receptors (*Mertk*), and bridging molecules (*Gas6* and *Pros1*) at 1, 3, and 7dpi (Fig. 2A–2E). To determine if Mertk protein is upregulated in Cx3cr1-expressing cells following CCI injury, we used *Cx3cr1*^*CreER*^ mice, which express EYFP in Cx3cr1-positive cells. Immunohistochemical analysis using Iba1 (a marker for microglia and PDM) revealed increased Mertk expression on IBA1+/Cx3cr1^EYFP+^ cells located in the peri-lesion at 3 dpi compared to the contralateral cortex (Fig. 2G–2O). To distinguish between microglia and PDMs, we used GFP bone marrow chimeric wild type mice. Quantification of Mertk was measured in serial coronal sections immuno-stained for IBA1 at 1 and 3 dpi. The estimated number of Mertk+/GFP+/IBA1+, PDMs and Mertk+/GFP−/IBA + microglia was measured in the ipsilateral cortex using non-biased stereology, optical fractionator probe, Stereoinvestigator. Consistent with scRNAseq data, Mertk expression was present on microglia and PDMs, however more microglia where positive for Mertk at 1dpi. Microglia expression remained consistent at 3dpi, however the estimated number of PDMs expressing Mertk was much higher. This could reflect the increased trafficking of monocytes to the brain at this time point (Fig. 2P–2Y). We show a distinct temporal change in expression of Mertk across two distinct myeloid populations following brain injury.

### Initiation of efferocytosis by Cx3cr1 + microglia/macrophages following CCI injury.

**Figure 3:**
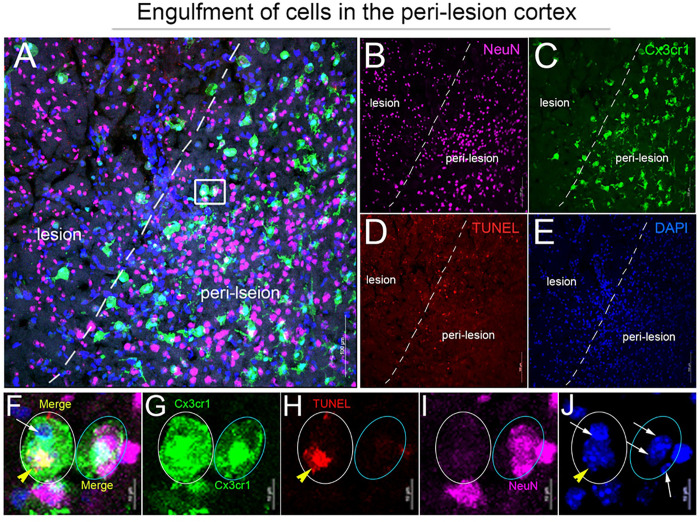
Engulfment of apoptotic neurons by Cx3cr1-expressing microglia/macrophages in the injured cortex following CCI. A-E) Representative confocal images for TUNEL (red, D)- and NeuN (purple, B)-stained coronal section of *Cx3cr1* ^*EYFP-CreER /+*^ mice at 3 dpi. F-J) Region of interest (ROI) showing Cx3cr1^+^ cells (green) containing TUNEL^+^ cells (yellow arrowhead) and NeuN+ cells (white arrow) in the peri-lesion cortex.

To determine if the change in Mertk expression in microglia and PDMs is correlated with the initiation of apoptotic neuron engulfment, coronal sections of CCI-injured *Cx3cr1*^*CreER*^ mice were stained with TUNEL and anti-NeuN antibody (Fig. 3). Z-stacked confocal images show Cx3cr1^EYFP+^ efferocytes containing DAPI+/TUNEL + and/or DAPI+/NeuN + nuclei along with DAPI+/NeuN−/TUNEL − nuclei in the peri-lesion cortex at 3 dpi (Fig. 3F–3J). This data provides the first evidence for the engulfment of dead/dying neurons by microglia and PDMs in traumatic brain injured mice.

### EphA4 deficiency in PDMs enhances the clearance of apoptotic debris in the damaged cortex following CCI injury.

**Figure 4:**
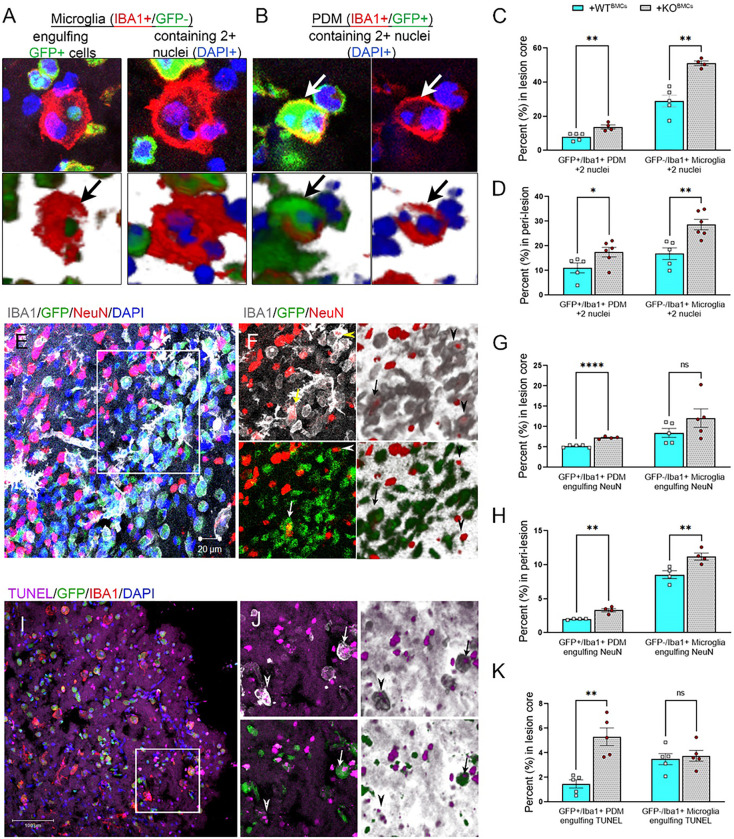
Peripheral-immune EphA4 deficiency enhances efferocytosis of PDMs and microglia in the damaged cortex following CCI injury. Serial coronal sections of WT ^+WT BMCs^ and WT^+KO BMCs^ mice were stained with IBA1 (red) (A-B), NeuN (red), and IBA1 (gray) (E, F), or TUNEL and IBA1 (I, J). A-B) Representative images showing microglia (IBA1+/GFP−) engulfing GFP+ cells (A, left) and containing 2+ nuclei (A, right) or PDM (IBA1+/GFP+) containing 2+ nuclei (B). C-D) quantification of microglia and PDM containing 2+ nuclei in the core and perilesion at 3dpi. E, F) Microglia (arrowhead) and PDM (arrow) engulfing NeuN+ neurons. G, H) Quantification of microglia and PDM engulfing NeuN+ neurons. I, J) Microglia (arrowhead) and PDM (arrow) engulfing TUNEL+ nuclei. K) quantification of microglia and PDM containing at least one TUNEL+ nuclei and one TUNEL− DAPI+ nuclei in the core at 3dpi. N=4–6 mice/group. Ns = non-significant; *P<0.05; **P<0.01; ***P<0.001, ****P<0.0001. Multiple t-test.

To evaluate the role of peripheral-derived EphA4 in this response we induced CCI injury to WT (WT^+ WTBMCs^) and EphA4 KO (WT^+ KOBMCs^) GFP BM chimeric mice and stained serial coronal sections with anti-IBA1, anti-NeuN, or TUNEL. The percentage of PDMs (GFP+/IBA1+) and microglia (GFP−/IBA+) engulfing other cells, including peripheral immune cells (cells contain 2 or more DAPI + nuclei including GFP cells; Fig. 4A–4D), neurons (cells contain at least one NeuN + cell and one DAPI + nuclei; Fig. 4E–4H), or TUNEL + cells (cells contain at least one TUNEL + and one TUNEL − nuclei; Fig. 4I–4K) was counted in the core and/or peri-lesion of the injured cortex. We found that the percentage of PDMs and microglia containing 2 + nuclei is significantly higher in the lesion core (Fig. 4C) and peri-lesion (Fig. 4D) of WT^+^ KOBMCs than WT^+ WTBMCs^. The percentage of PDMs engulfing NeuN + cells in the lesion core (Fig. 4G) and the peri-lesion (Fig. 4H), as well as TUNEL + cells in the lesion core (Fig. 4K), is higher in WT^+ KOBMCs^ than WT^+ WTBMCs^. Microglia engulfing NeuN + cells are significantly higher in the peri-lesion of WT^+ KOBMCs^ compared to WT^+ WTBMCs^. No significant difference was observed in microglia engulfing NeuN + or TUNEL + cells in the lesion core. This data suggests that EphA4 deficiency in BM-derived immune cells enhances the efferocytosis capacity of PDMs and peri-lesional microglia.

### Deficiency of peripheral-derived EphA4 enhances ERK/Stat6/Mertk signaling in the CCI-injured cortex.

**Figure 5:**
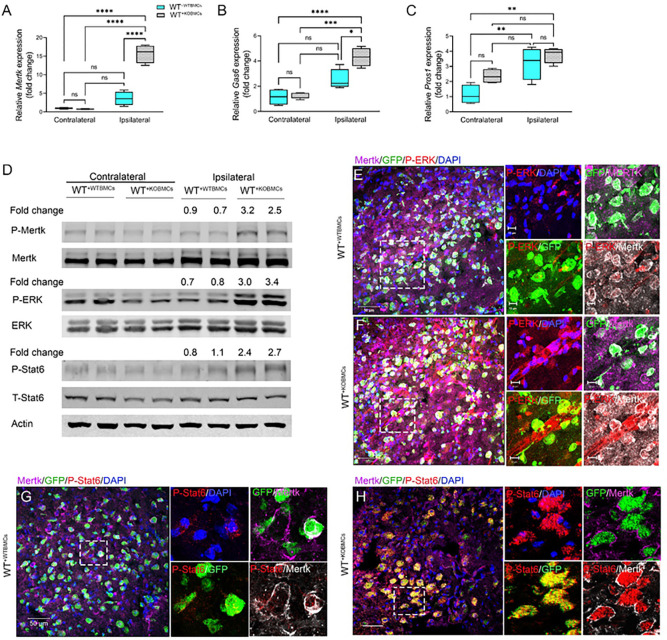
Peripheral EphA4 deficiency promotes P-ERK/P-Stat6/Mertk signaling. A-C) mRNA expression of the efferocytosis receptor (Mertk, A) and its ligands (Gas6, B) and (Pros1, C) in the contralateral and ipsilateral cortex of WT^+WT BMCs^ and WT^+KO BMCs^ mice at 3dpi. N=5–6 mice/group. Ns = non-significant; *P<0.05; **P<0.01; ***P<0.001, ****P<0.0001. Two-way ANOVA followed by Šídák’s multiple comparisons test. D) Western blot analysis shows increased Mertk, ERK, and Stat6 phosphorylation in the ipsilateral cortex of chimeric WT^+KO BMCs^ mice. E-H) Representative images showing peripheral-derived GFP+ cells expressing Mertk (purple) and P-ERK (E, F) and P-Stat6 (G, H) in WT^+WT BMCs^ and WT^+KO BMCs^ mice.

Given that myeloid Mertk-mediated efferocytosis signaling may predominant in the brain following CCI injury and is enhanced in the absence of EphA4 (Figs. 1 & 2), we sought to investigate the mRNA and protein phosphorylation status of key players in this pathway. We found that mRNA expression of *Mertk* and *Gas6* was significantly higher in the ipsilateral cortex of WT^+ KOBMCs^ at 3dpi when compared to WT^+^ WTBMCs (Fig. 5A, 5B). No significant difference was observed in *Pros1* transcript (Fig. 5C). Further, Western blot analysis showed increased phosphorylation of Mertk (P-Mertk), ERK 1/2 (P-ERK), and Stat6 (P-Stat6) in the ipsilateral cortex of WT^+ KOBMCs^ at 3 dpi compared to WT^+ WTBMCs^ (Fig. 5D & Supp. Figure 1). To specifically address whether ERK and Stat6 are present on Mertk expressing cells, coronal sections were immune-stained with anti-Mertk and anti-P-ERK (Fig. 5E & 5F) or anti-Mertk and anti-P-Stat6 (Fig. 5G & 5H). Confocal images show colocalization of P-ERK and P-Stat6 with MERK in the ipsilateral cortex of WT^+ KOBMCs^ mice and that Stat6 is increased on GFP expressing cells in KO mice compared to WT. This indicates the co-expression of these key signaling molecules on infiltrating efferocytes.

### Enhanced efferocytosis in EphA4-deficient bone marrow-derived macrophages (BMDMSs) is mediated by ERK/Stat6 pathway.

**Figure 6:**
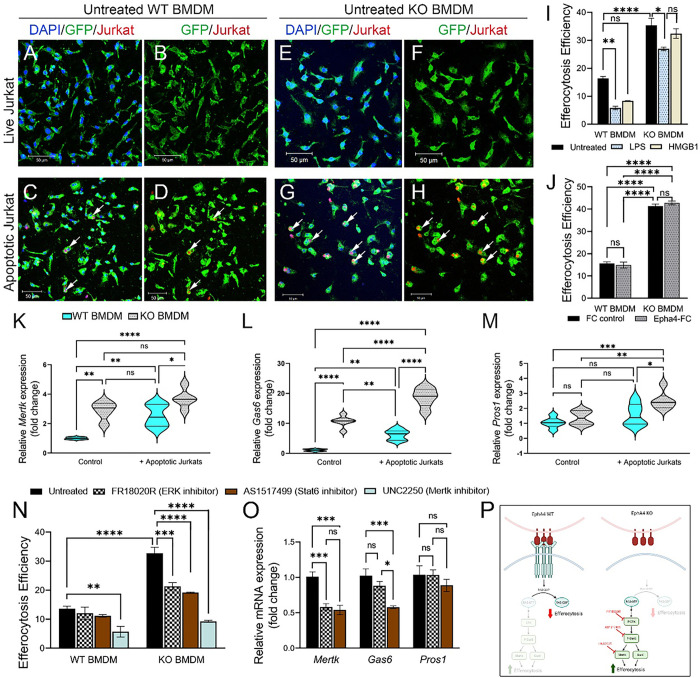
EphA4-null BMDMs show enhanced efferocytosis that is regulated by ERK/ Stat6/Mertk. A-I) EphA4 deletion enhances the efferocytosis efficiency of BMDMS *in vitro*. A-H) Representative images showing the engulfment of the pHrodo-stained apoptotic (but not live) Jurkat cells (red) by GFP+ untreated WT (A-D) and EphA4 KO (E-H) BMDMS. I) Quantification of the efferocytosis efficiency of WT and EphA4 KO BMDMS after stimulation with LPS and HMGB1 for 4 hours. J) Efferocytosis of EphA4 KO BMDMSs is mediated by the blockade of forward EphA4, not reverse ephrin signals. Treatment of WT and EphA4 KO BMDMS with clustered EphA4-FC to activate reverse ephrin signals did not reduce the efferocytosis of EphA4 KO BMDMS. K-M) mRNA expression of Mertk (K), Gas6 (L), and Pros1 (M) with and without engulfment of apoptotic Jurkat cells. EphA4 KO BMDMSs have higher expression of Mertk and Gas6 than WT. N) The use of Mertk inhibitor reduced efferocytosis of both WT and EphA4 KO BMDMS; however, Stat6 and ERK inhibitors selectively reduced efferocytosis in EphA4 KO BMDMS. O) Stat6 inhibitor reduced Mertk and Gas6 expression, and ERK inhibitor reduced Gas6 expression in KO BMDMS engulfing apoptotic Jurkat cells. P) Suggested pathway for the regulation of efferocytosis by EphA4. N=5–6 mice/group. Ns = non-significant; *P<0.05; **P<0.01; ***P<0.001, ****P<0.0001. Two-way ANOVA followed by Šídák’s multiple comparisons test (I-N) or one-way ANOVA followed by Tukey’s multiple comparisons test (O).

To confirm that EphA4 deficiency in bone marrow-derived macrophages enhances the clearance of apoptotic debris, an *in vitro* efferocytosis assay was performed. GFP + BMDMSs from *Epha4*^*+/+*^*/ROSA*^*mTmG*^*/Tie2-Cre* (WT) or *Epha4*^f/f^/ *ROSA*^*mTmG*^*/Tie2-Cre* (KO) mice were co-cultured with pHrodo-stained live or apoptotic Jurkat cells. Apoptosis was induced by treating Jurkat cells with staurosporine. After 3 hours of treatment, 90% of Jurkat cells were in the early phase of apoptosis (Annexin V + and PI^−^, Supp. Figure 2). No engulfment of live Jurkat cells was observed in EphA4 WT or EphA4 KO macrophages (Fig. 6A–6H). More engulfment of apoptotic Jurkat cells was observed in untreated, LPS-treated, and HMGB1-treated EphA4 KO macrophages when compared to WT (Fig. 6I). Importantly, treating macrophages with EphA4-FC clusters (to activate the reverse Ephrin signaling) did not reduce the efferocytosis efficiency of EphA4 KO macrophages confirming the enhanced efferocytosis in KO macrophages is mediated by the blockade of EphA4 forward signals (Fig. 6J). In addition, Mertk and Gas6 expression is significantly upregulated in EphA4 KO macrophages in the absence of apoptotic Jurkat cells compared to WT macrophages. After engulfing apoptotic Jurkat cells, Mertk and Gas6 expression increased in WT and KO macrophages; however, the expression is significantly higher in KO than in WT macrophages (Fig. 6K, 6L). Pros1 expression increased only in EphA4 KO macrophages after apoptotic Jurkat cell engulfment (Fig. 6M). To determine if enhanced efferocytosis in EphA4 KO macrophages is mediated by Mertk, ERK, and Stat6 activation, selective inhibitors were used. Mertk inhibitor (UNC2250) reduced efferocytosis efficiency of both WT and KO macrophages; however, ERK inhibitor (FR18020R) and Stat6 inhibitor (AS1517499) selectively reduced efferocytosis efficiency of EphA4 KO macrophages (Fig. 6N). Interestingly, ERK inhibition reduced Mertk expression in EphA4 KO macrophages engulfing apoptotic cells, and Stat6 inhibition reduced Mertk and Gas6 expression. A non-significant difference was observed in Pros1 expression in the presence of ERK or Stat6 inhibitors (Fig. 6O). Data suggests that EphA4 forward signaling reduces macrophage efferocytosis by inhibiting ERK and Stat6 activation, which in turn reduces the expression of efferocytosis receptor (Mertk) and its ligand (Gas6) and restricts the efferocytosis process (Fig. 6P).

### Peripheral EphA4 deficiency reduced apoptosis and improved functional outcomes following CCI injury.

**Figure 7:**
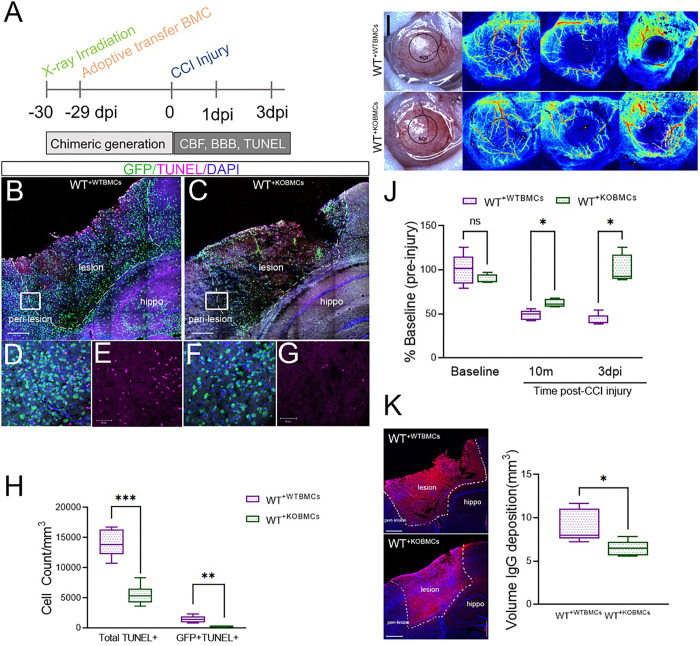
Peripheral EphA4 deficiency reduces cortical cell death and improves cerebral blood flow and BBB stability after CCI injury. A) Experimental outline. B-H) Apoptotic TUNEL+ (Purple) cell count is reduced in WT^+KO BMCs^ (B, D, E) compared to WT^+WT BMCs^ (C, F, G) mice. The total number of TUNEL+ and GFP+/TUNEL+ cells (H) was counted using image J in 3 serial sections using 5×7 tiled z-stack confocal images. I, J) Cerebral blood flow is improved in WT^+KO BMCs^ compared to WT^+WT BMCs^. Blood flow was measured using laser speckle contrast imaging (LSCI) at 10 minutes and 3 days post-injury and presented as a percentage of the baseline. K) Blood-brain barrier permeability is improved in WT^+KO BMCs^ compared to WT^+WT BMCs^. The volume of IgG deposition was measured at 3 dpi in serial coronal sections using Cavalieri Estimator from StereoInvestigator. N=5–6 mice/group. Ns = non-significant; *P<0.05; **P<0.01; ***P<0.001. Multiple t-test (I, H) or multiple t-test (K).

To determine if improved efferocytosis in bone marrow chimeric EphA4 KO mice is associated with reduced apoptotic cell accumulation, the number of apoptotic cells was counted in serial coronal sections stained with TUNEL at 3 dpi using the optical fractionator probe function on Stereoinvestigator (Fig. 7A). The total number of apoptotic cells (total TUNEL+) and apoptotic peripheral-derived immune cells (GFP + TUNEL+) were significantly reduced in WT^+ KOBMCs^ compared to WT^+ WTBMCs^ mice (Fig. 7B–7H). This effect was correlated with improved cerebral blood flow and reduced blood-brain barrier permeability. Cerebral blood flow was measured at 10 minutes and 3dpi using Laser Speckle Contrast Imaging (LSCI) and calculated as percentage flow from the baseline (pre-injury). WT^+ KOBMCs^ showed a significant increase in cerebral blood flow at 3dpi compared to WT^+ WTBMCs^ (Fig. 7I, 7J). Blood-brain barrier permeability was measured at 3 dpi by estimating the volume of IgG deposition in serial coronal section using the Cavalieri Estimator from StereoInvestigator software. Significant reduction in IgG deposition was observed in WT^+^ KOBMCs compared to WT^+ WTBMCs^ indicating improved blood-brain barrier function and neuroprotection (Fig. 7K). We find greater improvements in functional outcome correlate with efferocytosis enhancement by gene deletion of EphA4 on infiltrating innate immune cells.

## Discussion

Apoptosis is a prominent mode of cell death that mediates tissue damage and may contribute to the propagation neuroinflammation following brain injury. In cases of excessive apoptotic cell death, an overwhelmed clearance mechanism may result in the accumulation of apoptotic cells and cellular debris, prolonging the exposure to pro-inflammatory signals and activated immune cells. Therefore, a greater understanding of the efferocytotic process may aid in the development of strategies targeting the timely removal of dying cells in the brain. Our results demonstrate an upregulation of “find-me” signal receptors (S1pr1 and Cx3cr1), engulfment receptor (Mertk), and bridging molecules (Gas6 and Pros1) in the damaged cortex, indicating the onset of efferocytosis acutely following trauma. We identified that resident (microglia) and peripheral-derived (monocyte/macrophages; PDMs) myeloid cells show distinct temporal expression patterns of Mertk. Notably, we find that ephrin type-A receptor 4 (EphA4) deficiency on infiltrating immune cells enhanced the capacity of PDMs and peri-lesional microglia for efferocytosis, as well as enhanced cortical expression of *Mertk*, and *Gas6* transcripts. Our investigation into the molecular mechanisms revealed increased phosphorylated (p) levels of p-Mertk, p-ERK and p-Stat6 in the injured cortex of chimeric EphA4 KO mice. In addition, inhibition of ERK and Stat6 attenuated the enhanced efferocytosis and *Mertk* expression in EphA4-deficient BMDMSs *in vitro*. This demonstrates that EphA4 suppresses efferocytosis by inhibiting the ERK/Stat6 pathway. These findings highlight a new and novel role for the axon guidance molecule EphA4 in regulating the coordinated process of efferocytosis, which may contribute to the overall neuroinflammatory milieu after brain injury.

EphA4 has been identified as a regulator of neuroinflammation and secondary injury following brain trauma. Our previous studies utilizing mouse models and bone marrow chimeric approaches have demonstrated that the absence or inhibition of EphA4 in peripheral-derived monocytes/macrophages results in neuroprotection, reduced cortical infiltration of monocytes/macrophages, and a shift in their gene profile from a proinflammatory to an anti-inflammatory state [10, 12]. These findings underscore the pivotal role of EphA4 in mediating the pro-inflammatory phenotypic state of PDM, thereby prompting further investigation into whether EphA4 hampers the adequate clearance of apoptotic debris by modulating the phenotypic state of efferocytes following CCI injury. Interestingly, deletion of EphA4 on infiltrating immune cells fosters the augmentation of efferocytosis in both PDMs and resident microglia. This may be due to the fact that EphA4-deficiency in monocytes enhances expression of *Gas6*, which may fuel the neighboring microglia and promote their efferocytotic abilities. This also suggests that monocytes may have key properties that may allow them to communicate with and regulate microglial function.

Eph receptors and ephrin ligands facilitate intercellular communication to regulate diverse processes, including adhesion, repulsion, migration, survival, proliferation, remodeling, and differentiation. Upon binding, the clustering of Eph receptors and ligands triggers a cascade of signaling events, in both the cells expressing the receptor and those bearing the ligand [19]. EphA4-null BMDMCs, treated with clustered EphA4-FC, showed no effect on efferocytosis efficiency confirming forward EphA4 signaling on monocyte/macrophages mediates the suppression of ERK/Stat6 signaling and subsequent enhancement of efferocytosis. Whether the genetic deletion of PDM EphA4 may impair reverse ephrin signaling on apoptotic cells and its relevance in the current study remains unknown and requires additional investigation. Forward Eph signaling exhibits a remarkable ability to attenuate the RAS-ERK pathway, overriding its activation by other receptor tyrosine kinases [19, 20]. This inhibition has been observed in diverse scenarios, including the modulation of growth cone motility in neurons [21] and the suppression of tumorigenicity in cancer cells [22, 23]. The mechanism underlying Eph receptor-dependent ERK inhibition often involves the activation of the RAS GTPase, p120 RAS GAP, which leads to the subsequent inactivation of H-RAS [24]. Conversely, Eph receptors can activate the RAS-ERK pathway in specific contexts, promoting cellular processes such as proliferation, early gene transcription, cell migration, or repulsion [25, 26]. Eph receptor signaling exerts a complex and context-dependent influence on the RAS-ERK pathway, however, our novel findings indicate that EphA4 suppresses this signaling to limit efferocytosis in the brain following injury.

ERK1/2 signaling modulates multiple aspects of efferocytosis and inflammation resolution, including the regulation of phagocytic receptor expression, cytoskeletal rearrangement, and the production of anti-inflammatory molecules [27, 28]. Activation of the ERK1/2 signaling pathway can enhance the expression of phagocytic receptors, including Mertk, Gas6, MFG-E8, and integrins (such as αvβ3) [29–31]. In addition, ERK1/2 signaling is implicated in promoting actin polymerization and cytoskeletal rearrangement, thereby facilitating the process of efferocytosis [32]. Importantly, ERK1/2 activation also regulates the production of anti-inflammatory mediators during efferocytosis, promoting the polarization of macrophages toward a pro-resolving phenotype, which contributes to sustained efferocytosis and the resolution of inflammation [33, 34]. One crucial transcription factor influenced by ERK1/2 activation to regulate macrophage polarization is Stat6 [35, 36]. The phosphorylation of Stat6 promotes the transcription of genes involved in macrophage pro-resolving and efferocytosis response, such as Gas6 and Mertk [37, 38]. In the present study, we observed an activation of Mertk, ERK1/2, and Stat6 in the injured cortex of chimeric EphA4 KO mice. Furthermore, selective inhibition of ERK and Stat6 reduced efferocytosis and Mertk expression, specifically in EphA4-deficient BMDMS *in vitro*. These findings suggest that EphA4 impedes efferocytosis by inhibiting the P-ERK/P-Stat6/Mertk signaling pathway.

## Conclusions

Efferocytosis occurs acutely in the brain following trauma in a limited capacity. Blockade of EphA4 receptor function improves this activity, which coincided with tissue protection, restoration of cerebral blood flow and BBB stability. Unraveling the molecular mechanisms underlying efferocytosis in TBI will led to new therapeutic avenues promoting this process and in order to mitigate the deleterious consequences of apoptotic death and facilitating optimal recovery following brain trauma.

## Figures and Tables

**Table 1:** Sequence of primers used in qRT-PCR

Target gene	Primer sequence 5′ → 3′

*GAPDH*	F: CGT CCC GTA GAC AAA ATG GT R: TCA ATG AAG GGG TCG TTG AT
*Mertk*	F: AAG TGG ATC GCC ATC GAG AG R: GGA GTC ATT CCC CGT GTT GT
*Gas6*	F: GCT TCG GTA CAA TGG CGT T R: GAC AAG GTT ACG TTG CAG CTC
*Pros1*	F: TTG GTG GAT TCT CGC TCT GG R: GAT TGC TGA TCC GAG CAC AG
*Cx3cr1*	F: GTG AGT GAC TGG CAC TTC CTG R: AAT AAC AGG CCT CAG CAG AAT C
*Axl*	F: TCA TGT GAA GCC CAC AAT GC R: ACC TCT AGC TCC GTA GGT TGT

## Data Availability

Data are available upon request.

## Abbreviations

AnnV: Annexin V
BM: Bone marrow
BMCs: Bone marrow cells
BMDMS: Bone marrow-derived macrophages
CCI: controlled cortical impact
Cx3cr1: CX3C motif chemokine receptor 1
EphA: Erythropoietin-producing hepatocellular carcinoma receptors type A
ERK 1/2: Extracellular signal-regulated kinase 1/2
Gas6: Growth arrest-specific 6
GFP: Green fluorescence protein
IBA1: Ionized calcium-binding adaptor molecule 1
KO: Knock out
LPS: Lipopolysaccharide
LSC: Laser speckle contrast
Mertk: Mer proto-oncogene tyrosine kinase
NeuN: Neuronal nuclei protein
PDMs: Peripheral-derived macrophages
PI: Propidium iodide
Pros1: Protein S
PtdSer: Phosphatidyl serine
S1pr1: Sphingosine-1-Phosphate Receptor 1
Stat6: Signal transducer and activator of transcription 6
STS: Staurosporine
TBI: Traumatic brain injury
WT: Wild-type
WT+KOBMCs: EphA4-knockout bone marrow chimeric mice
WT+WTBMCs: Wild-type bone marrow chimeric mice
